# Global profiling of alternative splicing landscape responsive to drought, heat and their combination in wheat (*Triticum aestivum* L.)

**DOI:** 10.1111/pbi.12822

**Published:** 2017-09-20

**Authors:** Zhenshan Liu, Jinxia Qin, Xuejun Tian, Shengbao Xu, Yu Wang, Hongxia Li, Xiaoming Wang, Huiru Peng, Yingyin Yao, Zhaorong Hu, Zhongfu Ni, Mingming Xin, Qixin Sun

**Affiliations:** ^1^ State Key Laboratory of Crop Stress Biology for Arid Areas College of Agronomy Northwest A&F University Yangling Shaanxi China; ^2^ State Key Laboratory for Agrobiotechnology Key Laboratory of Crop Heterosis Utilization (MOE) Beijing Key Laboratory of Crop Genetic Improvement China Agricultural University Beijing China

**Keywords:** Alternative splicing, Polyploidy, Heat, Drought, Transcriptome, Wheat

## Abstract

Plant can acquire tolerance to environmental stresses *via* transcriptome reprogramming at transcriptional and alternative splicing (AS) levels. However, how AS coordinates with transcriptional regulation to contribute to abiotic stresses responses is still ambiguous. In this study, we performed genome‐wide analyses of AS responses to drought stress (DS), heat stress (HS) and their combination (HD) in wheat seedlings, and further compared them with transcriptional responses. In total, we found 200, 3576 and 4056 genes exhibiting significant AS pattern changes in response to DS, HS and HD, respectively, and combined drought and heat stress can induce specific AS compared with individual one. In addition, wheat homeologous genes exhibited differential AS responses under stress conditions that more AS events occurred on B subgenome than on A and D genomes. Comparison of genes regulated at AS and transcriptional levels showed that only 12% of DS‐induced AS genes were subjected to transcriptional regulation, whereas the proportion increased to ~40% under HS and HD. Functional enrichment analysis revealed that abiotic stress‐responsive pathways tended to be highly overrepresented among these overlapped genes under HS and HD. Thus, we proposed that transcriptional regulation may play a major role in response to DS, which coordinates with AS regulation to contribute to HS and HD tolerance in wheat.

## Introduction

Global agriculture is facing dramatic climatic changes including extreme high temperature and intense drought stress, which often occur simultaneously under natural environment (Prasad *et al*., 2011). These are main factors severely limiting crop growth and causing dramatic yield loss, particularly for chimonophilous wheat, which serves as the staple food source for more than 30% of the human population (IWGSC, 2014). To cope with various environmental stresses, plant has evolved complex systems to achieve enhanced tolerance. Therefore, understanding the complicated responses of wheat to heat, drought and their combined stresses is valuable to improve yield and quality potential in breeding programmes.

Alternative splicing (AS) refers to pre‐mRNA processing events that multiple mature mRNAs arise from a single gene locus due to alternate splice site choices, including intron retention (IR), exon skipping (ES), alternative 5′ splicing site (Alt5′SS) and alternative 3′ splicing site (Alt3′SS) (Syed *et al*., 2012). AS often incorporates premature termination codons (PTCs) into alternate transcripts which are subsequently degraded by non‐sense‐mediated decay (NMD) or produce truncated proteins (Kalyna *et al*., 2012; Ottens and Gehring, 2016). Alternatively spliced transcripts can also result in proteins with altered structures, functions or subcellular locations (Gracheva *et al*., 2011; Kriechbaumer *et al*., 2012). In addition, some alternate protein variants can compete with normal variants and interfere their functions in a dominant negative manner (Pose *et al*., 2013; Seo *et al*., 2011). Thus, AS has been documented to play important regulatory roles at post‐transcriptional level in response to hostile environments in plants (Reddy *et al*., 2013; Syed *et al*., 2012).

Genome‐wide analyses of gene expression profiling in response to heat (HS), drought (DS) and heat and drought combined stresses (HD) have been widely investigated in plants, and thousands of stress‐responsive genes enriched in diverse biological functions were identified, for example Heat Shock Factors (HSFs), Heat Shock Proteins (HSPs) and Drought Responsive Elements Binding Factors (DREBs) (Johnson *et al*., 2014; Liu *et al*., 2015; Rizhsky *et al*., 2004; Szucs *et al*., 2010). However, how alternative splicing modulates transcriptome responses to HS, DS and HD together with transcriptional regulation is still ambiguous. Recently, emerging evidences have been reported that AS has extensive biological relevance conferring stress tolerance in plants. For example, in Arabidopsis and rice (*Oryza sativa*), conserved alternative splicing of *HSFA2* introduces a PTC into splicing isoform *HSFA2‐II* and leads to the production of truncated proteins without transcriptional activation activity under normal condition (Cheng *et al*., 2015; Staiger and Brown, 2013; Sugio *et al*., 2009). However, the expression of alternative splice isoform *HSFA2‐I,* encoding a full‐length protein with transcription activation activity, is dramatically induced by HS, which triggers the activation of HS‐responsive genes in Arabidopsis and rice under heat stress condition (Cheng *et al*., 2015; Sugio *et al*., 2009). Another key abiotic stress regulator *DREB2B* was also reported to be modulated by AS in rice (Matsukura *et al*., 2010). *OsDREB2B1*, the nonfunctional isoform containing a PTC introduced by an exon inclusion, is the dominant isoform under normal conditions. However, the functional isoform *OsDREB2B2*, encoding a protein with transcriptional activation activity, was dramatically induced to promote the expression of downstream target genes and enhance stress tolerance under HS and DS conditions (Matsukura *et al*., 2010). Interestingly, wheat *WDREB2*, an ortholog of *OsDREB2B*, exhibited similar AS pattern under low temperature, drought and salt stress, highlighting the conservation of AS modulation among plant species (Egawa *et al*., 2006; Terashima and Takumi, 2009).

Supporting by high‐throughput sequencing platform, genome‐wide profiling of AS events in plant species revealed that ~33%–60% mRNA are alternatively spliced (Li *et al*., 2014; Marquez *et al*., 2012; Shen *et al*., 2014; Thatcher *et al*., 2014; Zhang *et al*., 2010). Further analysis found that ~4%–7% splicing isoforms specifically occur when subjected to drought and salt stresses in grape (*Vitis vinifera*) leaf and root (Vitulo *et al*., 2014). In maize (*Zea mays*), 1060 and 932 DS‐induced AS events were identified in leaf and ear, accounting for 6.2% and 5.5% of expressed genes, respectively (Thatcher *et al*., 2016). Genome‐wide investigation of AS in moss (*Physcomitrella patens*) has identified 1779 AS events significantly responsive to elevated temperature, accounting for nearly half of expressed genes (Chang *et al*., 2014). Furthermore, approximately 7500 genes with HS‐dependent accumulation of IR and ES were detected in tomato (*Solanum lycopersicum*) pollen, including six *HSFs* and 29 *HSPs*, which play key roles in plant heat shock response (Keller *et al*., 2016). However, comprehensive comparison of AS responsive to heat, drought and their combination is still not available in plant. And it is barely known how AS and transcriptional regulation cooperate to modulate transcriptome reprogramming in response to environmental stresses.

In this study, genome‐wide profiling of alternative splicing was performed on wheat seedlings subjected to DS, HS and HD, which was further compared with transcriptional changes to investigate their correlations under stress conditions. Moreover, we examined the differences in AS landscape across A, B and D subgenomes of wheat in response to DS, HS and HD. Our results indicated that homeologous genomes exhibited differential AS responses to abiotic stresses, and gene expression and AS level regulations can work together to orchestrate the transcriptional and post‐transcriptional reprogramming in response to HS and HD, whereas they might function independently in DS response.

## Results

### Identification of alternative splicing events in wheat seedlings

**Figure 1:**
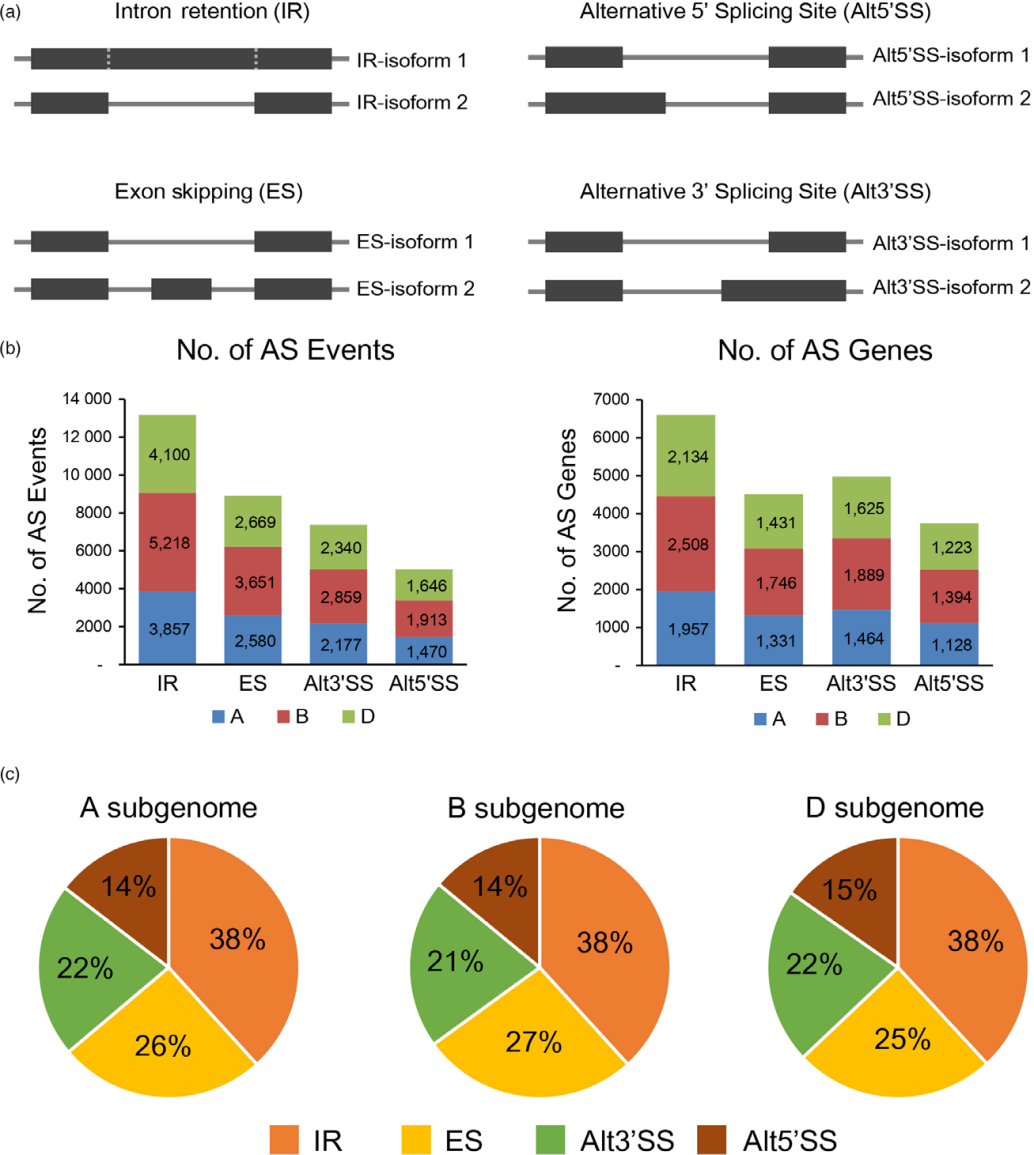
Summary of alternative splicing events in wheat seedlings. (a) Schematic representation of four types of alternative splicing (AS): intron retention (IR), exon skipping (ES), alternative 5′ splicing site (Alt5′SS) and alternative 3′ splicing site (Alt3′SS). Each event refers to two types of isoforms named isoform 1 and isoform 2, which can be distinguished and qualified with junction supporting reads. (b) The number of different types of AS events and the corresponding AS genes identified in wheat seedlings. The AS events and genes identified in different subgenomes are indicated by different colours (blue, red and green for A, B and D subgenomes, respectively). (c) Distributions of different modes of AS events in A, B and D subgenomes.

To investigate the transcriptome responses to environmental stresses, we performed RNA‐Seq experiments on wheat seedlings subjected to drought stress (DS), heat stress (HS) and their combination (HD) at 1 h and 6 h. Our previous study showed that thousands of genes involved in diverse functions were differentially expressed in response to these stresses (Liu *et al*., 2015). Here, we further examined the comprehensive profiling of AS landscape under these stress conditions using the same RNA‐Seq data (Table S1). Briefly, high‐quality reads were firstly mapped to wheat reference genome and AS events were identified by junction‐mapped reads as described by previous studies (Figure S1, Experimental procedures) (Chang *et al*., 2014; Ling *et al*., 2015). Finally, 34 480 AS events, corresponding to 12 709 genes, were identified before and/or after stress treatments, accounting for ~24.6% of expressed genes in wheat seedlings (Figure 1a, b). Specifically, 3837, 4695 and 4177 AS genes were determined on subgenomes A, B and D, accounting for 23.3% (16 498), 25.9% (18 118) and 24.4% (17 111) of expressed genes, respectively. The distributions of AS types in A, B and D subgenomes are comparable; that is, IR was the most abundant (38%) AS events, followed by ES (25%–27%), Alt3′SS (21%–22%) and Alt5′SS (14%–15%), which was consistent with previous reports (Figure 1c) (Chang *et al*., 2014; Ding *et al*., 2014).

### Identification of stress‐responsive AS events in wheat seedlings

**Figure 2:**
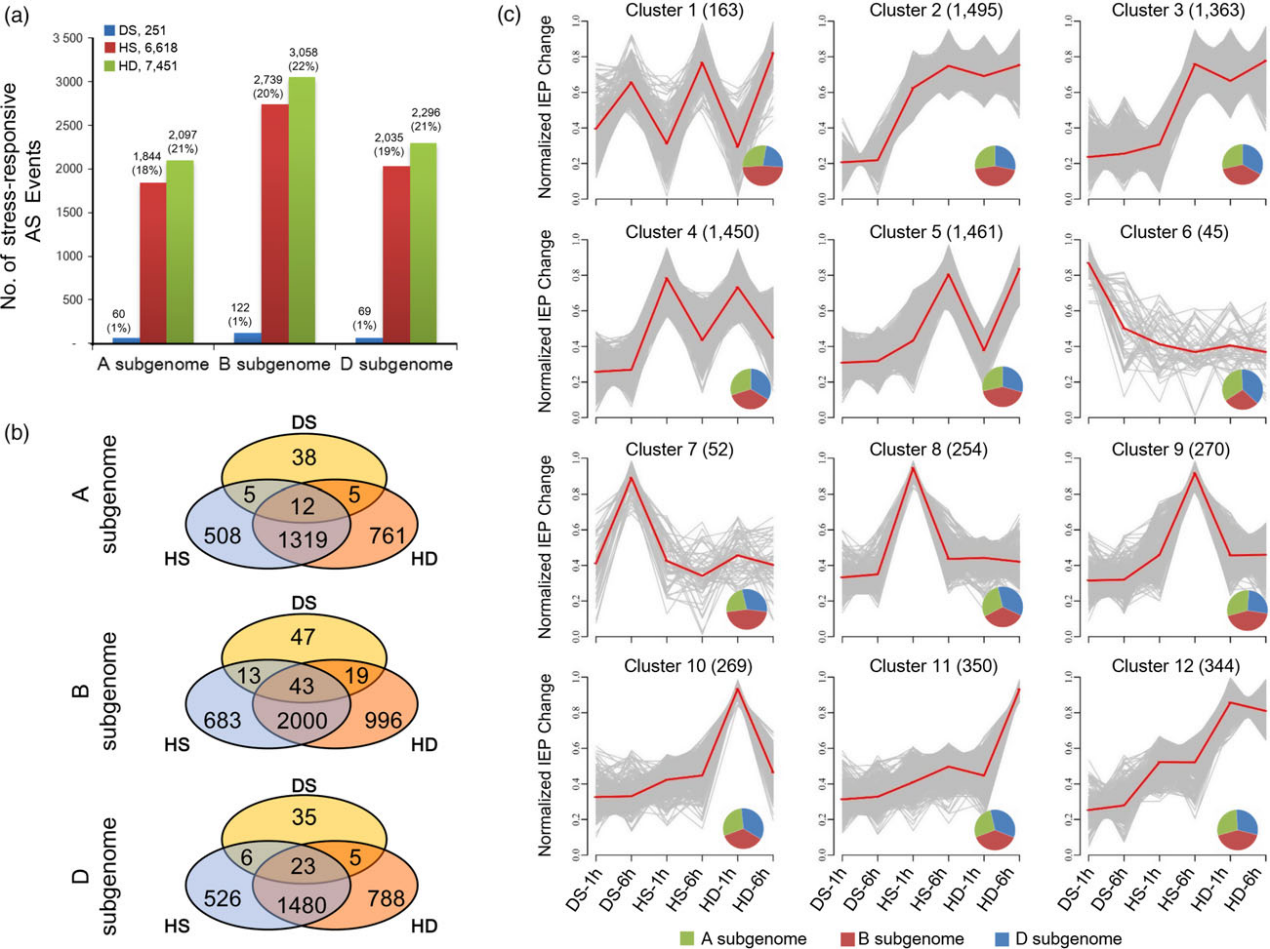
Identification and comparison analysis of stress‐responsive AS events in wheat seedlings. (a) The number of stress‐responsive AS events identified in A, B and D subgenomes under drought stress (DS), heat stress (HS) and their combination (HD) in wheat seedlings. The percentage in brackets represents the proportion of stress‐responsive AS events in totally identified ones in each subgenome. (b) Comparison of DS‐, HS‐ and HD‐responsive AS events in A, B and D subgenomes. (c) Clustering analysis of stress‐responsive AS events according to their isoform expression percentage (IEP = EXP_isoform1_/(EXP_isoform1_ + EXP_isoform2_)) variations after 1 h and 6 h of DS, HS and HD treatments. IEP variations represent the magnitude of AS pattern changes after stress treatments. Stress‐responsive AS events were clustered into 12 groups based on their IEP variations between control and each stress condition. The numbers in parentheses indicate the number of AS events in each cluster. *X*‐axis represents different stress treatments, and *y*‐axis represents centralized and normalized absolute IEP value changes. The red lines represent the mean IEP change trends of all AS events (grey lines) in each cluster. Venn diagrams in each plot indicate the distributions of AS events identified in A, B and D subgenomes for each cluster.

Stress‐responsive AS events referred to splicing isoforms of a gene exhibiting differential expression changes after stress treatments. Here, stress‐responsive AS events were defined by criteria ‘junction reads based FDR <0.05’ and ‘≥30% variation of isoform expression percentage (IEP, calculated as EXP_isoform1_/[EXP_isoform1_ + EXP_isoform2_]) change’ after stress treatments (Experimental procedures). Finally, 9312 stress‐responsive AS events were identified, including 251, 6618 and 7451 responsive to DS, HS and HD, respectively, which indicated stronger AS responses were induced by HS and HD compared with DS (Figure 2a, Data S1). Of these AS events, 5659 occurred to genes annotated as protein‐coding genes, among which, 2192, presented in CDS, were predicted to introduce PTCs, which might lead to non‐sense‐mediated transcript decay or produce truncated proteins; 1931 appeared in UTR regions which might result in translation efficiency alteration, whereas the other 1536 events emerged in CDS can lead to protein sequence changes (Data S2).

Further analysis showed that more stress‐responsive AS events were identified in B subgenome compared to A and D. Specifically, 122 (0.9%), 2739 (20.1%) and 3058 (22.4%) AS events were characterized responsive to DS, HS and HD on subgenome B, whereas the corresponding number for genomes A and D was 60 (0.6%), 1844 (18.3%) and 2097 (20.8%) as well as 69 (0.6%), 2035 (18.9%) and 2296 (21.3%), respectively (Figure 2a). In addition, comparison of stress‐responsive AS events showed that 50.9%–54.4% (1331–2043) AS events were shared between HS and HD among three wheat homeologous subgenomes, whereas only 0.8%–2.0% (17–62) were overlapped between DS and HS/HD, indicating that HD‐induced AS responses were similar to HS, but distinct from DS (Figure 2b). Next, we examined whether wheat homeologous genes exhibiting partitioned AS responses to these three stresses. To achieve this object, we first selected homeologous triplets which designed as gene loci that had exactly one representative member from each subgenome as described in previous reports (International Wheat Genome Sequencing Consortium, 2014). Then, triplets with all three homeologous genes being expressed were retained for further analysis (3499 triplets, 3499 × 3 = 10 497 homeologs). Among these triplets, 23, 264 and 292 were found having only one or two homeologs being differentially spliced under DS, HS and HD, suggesting the partitioned AS responses occurred to homeologous genes when subjected to abiotic stresses, although a proportion of triplets showed consistent alternative splicing among three homeologous genes (Figure S2).

Subsequently, we performed clustering analysis of IEP changes for all stress‐responsive AS events using Mfuzz program (Figure 2c, Data S3) (Kumar and E Futschik, 2007). As shown in Figure 2c, these AS events were classified into 12 groups according to their AS change profiles. Cluster 1, consisting of 163 AS events, showed responses to all three stress treatments. Clusters 2–5, representing a total of 5769 AS events, showed significant AS pattern changes under both HS and HD conditions, which highlights the similarity of AS responses induced by HS and HD (Figure 2c). Clusters 6–12 represented AS events specifically responsive to DS, HS or HD. Notably, Clusters 10–12 (963 AS events) only exhibited significant AS pattern changes in response to HD other than HS and DS, suggesting that combined heat and drought stress can induce specific AS responses (Figure 2c). Intriguingly, among these groups, subgenome B contributed to a significant bias in Clusters 1, 2 and 7 (accounting for 48%, 44% and 46%, respectively), whereas the three subgenomes showed a balanced contribution in other clusters (Figure 2c). Taken together, our results confirmed that alternative splicing events extensively occurred in all three subgenomes of hexaploid wheat in response to DS, HS and HD, and a proportion of homeologous triplets underwent partitioned AS modulation.

### Various abiotic stress‐responsive genes were subjected to AS modulation under DS, HS and HD

Genes containing stress‐responsive AS events were defined as differentially spliced genes (DSGs). Totally, 4821 DSGs (200, 3576 and 4056 under DS, HS and HD treatments, respectively), including various important abiotic stress‐responsive genes, were identified in wheat seedlings (Figure S3, Data S1). For instance, eight *HSF* genes, key regulators in plant heat shock response, exhibited significant AS pattern changes in response to HS and HD, including *TaHSFA2* (XLOC_044199) (Figures S3 and S4). Similar to *HSF*s, 12 *HSP* genes were found to be differentially spliced after DS, HS and HD treatments (Figure S3, Data S1). Moreover, many previously reported stress‐responsive genes, for example *AL5*,* WDR5A* and *TNO1*, were found exhibiting HD‐specific AS responses (Figure S3, Data S1) (Kim and Bassham, 2011; Liu *et al*., 2017; Wei *et al*., 2015). As *Ser/Arg‐rich* (*SR*) proteins were demonstrated to be key AS regulators in plants (Reddy and Ali, 2011), we further investigated the expression and alternative splicing changes in wheat *SR* genes in response to DS, HS and HD treatments. Totally, 19 and 18 *SR* genes were found to be differentially spliced under HS and HD conditions, whereas only one *SR* gene showed responsive AS pattern under DS (Figure S5a). In addition, the number of differentially expressed *SR* genes in response to HS and HD was also more than DS, with 24 and 26 responsive to HS and HD in comparison with five to DS (Figure S5b). More interestingly, the majority of SR genes (75%) exhibited higher expression levels in response to HS and HD than DS (Figure S5c).

**Figure 3:**
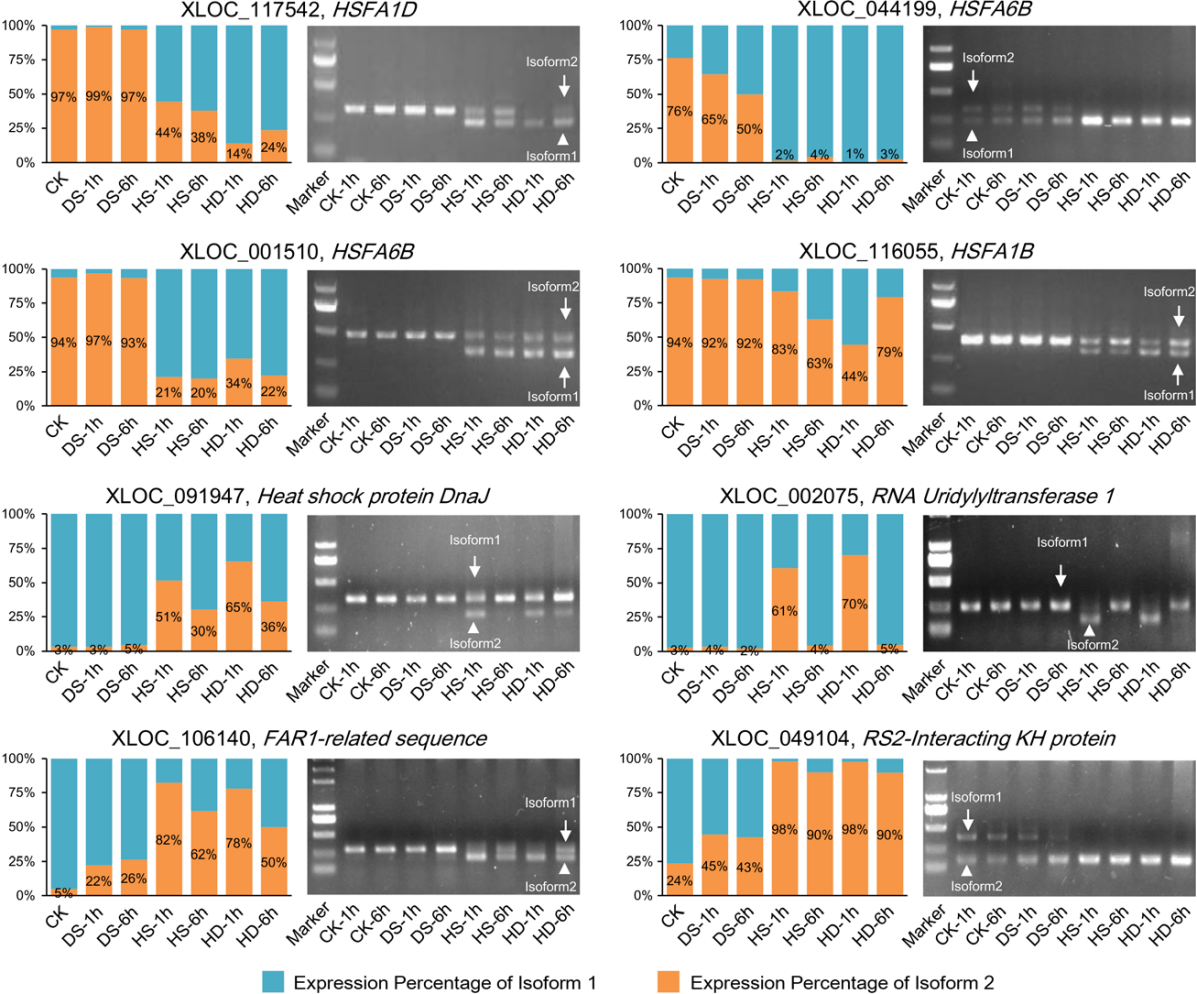
Experimental validation of stress‐responsive AS events by reverse transcription (RT)‐PCR in wheat seedlings. The bar charts show relative expression level of alternatively spliced isoform 1 (blue) and isoform 2 (yellow) of AS genes under control (CK), DS, HS and HD conditions revealed by RNA‐Seq. The RT‐PCR validation confirmed the computational prediction of AS events including five IR (XLOC_106140, XLOC_049104, XLOC_002075, XLOC_091947 and XLOC_116055), two ES (XLOC_044199 and XLOC_117542) and one Alt5′SS (XLOC_001510), which showed consistent results with RNA‐Seq. The primer pair used in each RT‐PCR allows the amplification of the two splice variants (isoforms 1 and 2).

To confirm the stress‐responsive AS events identified by RNA sequencing, we further validated the predicted AS patterns by reverse transcription (RT)‐PCR (Figure 3). The conserved primer pairs were used to amplify both splice variants (isoforms 1 and 2) in a single reaction. As expected, eight examined DSGs, including four *HSF* genes and one *HSP* gene, showed consistent AS patterns with their profiles revealed by RNA‐Seq data, which further confirmed the accuracy of our bioinformatic analysis (Figure 3). For example, the isoform 2 of *HSFA1D* (XLOC_117542) was only expressed under normal and DS conditions, while its isoform 1 was dramatically induced by HS and HD based on RNA sequencing (Figure 3). Likewise, we can only detect the isoform 2 band of *HSFA1D* under normal and DS conditions, while both isoform 1 and 2 bands were able to be amplified under HS and HD conditions (Figure 3).

### Comparative analysis of differentially expressed and differentially spliced genes

**Figure 4:**
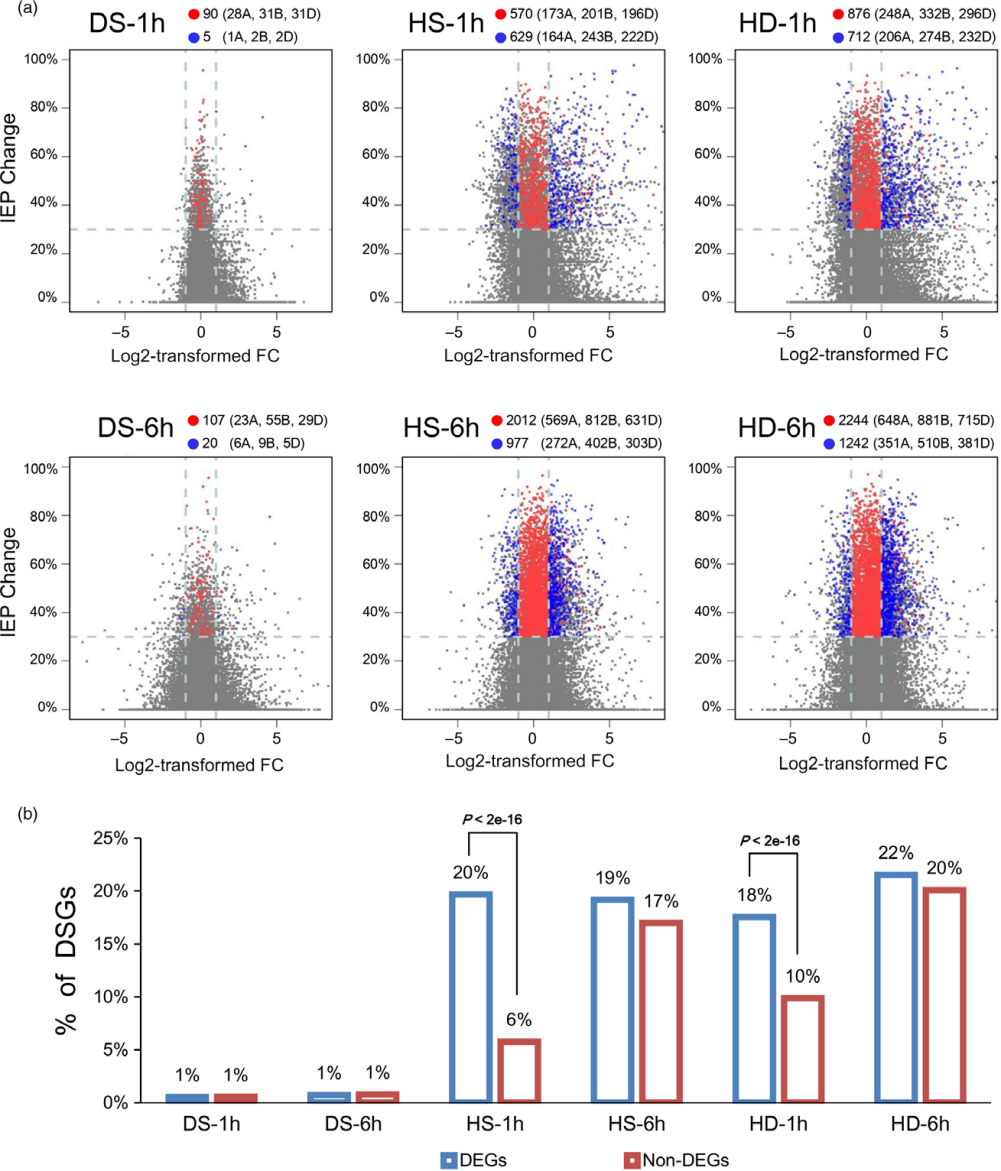
Comparison analysis of differentially spliced (DSGs) and differentially expressed genes (DEGs) in response to DS, HS and HD in wheat seedlings. (a) Scatter plots show alternative splicing pattern variations and gene expression abundance changes at 1 h and 6 h after DS, HS and HD treatments. The gene expression changes are represented by log2‐transformed fold changes (FC) of gene expression abundance, which are plotted on *x*‐axis. Splicing pattern variations are represented by the IEP changes between stress and control conditions for each gene, which are plotted on *y*‐axis. Horizontal dot lines indicate the threshold of ‘IEP change ≥30%’ used in DSGs identification, and vertical dot lines indicate the threshold of ‘LogFC ≥1 or ≤−1’ used in DEGs identification. Red dots indicate genes identified as DSGs specifically, blue dots indicate genes identified as both DSGs and DEG, and grey dots indicate the other genes expressed in wheat seedlings. The number of DSG‐specific (red dots) and DSG&DEG‐overlapped genes (blue dots) was indicated in each plot, and the numbers in brackets indicate the corresponding gene numbers in A, B and D subgenomes. (b) Comparison of the proportion of DSGs identified in DEGs and non‐DEGs under each stress condition. To avoid bias, only genes with more than 10 junction supporting reads were considered for the analysis. The proportion of DSGs in DEGs is much higher than in non‐DEGs under HS‐1 h and HD‐1 h, and the *P*‐values indicate significance levels based on Fisher's exact test.

To investigate the relationship between AS and transcriptional regulation, we further compared the genes undergoing AS and transcriptional changes in response to heat and drought stresses in wheat seedlings (Figure 4a). Only 24 DSGs (12% of all DS‐responsive DSGs) were found exhibiting differential expression patterns (identified as DEGs, Figure S6, Data S4) under DS conditions (Figure 4a, represented by blue dots). Whereas under HS and HD conditions, the number of DSGs that were also identified as DEGs increased to 1448 (40%) and 1696 (42%) (Figure 4a, represented by blue dots). In addition, 176, 2128 and 2360 DSG‐specific genes were identified in response to DS, HS and HD, respectively, suggesting that these genes were mainly subjected to AS modulation during heat and drought responses (Figure 4a, represented by red dots). Collectively, these results showed that there was very little overlap between DSGs and DEGs during DS response, whereas much more under HS and HD conditions, which indicated that AS can work together with transcriptional regulation to contribute to HS and HD responses.

To examine whether the AS change can be affected by transcriptional activity during HS and HD responses in wheat, we compared the proportion of DSGs in differentially (DEGs) and non‐differentially expressed genes (non‐DEGs), and found that the proportion of DSGs in DEGs is higher than that in non‐DEGs after 1 h of HS and HD treatments (20% vs 6% for HS‐1 h, 18% vs 10% for HD‐1 h) (Figures 4b and S7). Additionally, the global IEP variations in AS were more intense in DEGs than that in non‐DEGs under HS‐1 h and HD‐1 h, suggesting that the AS response might be associated with transcriptional activity change under these two conditions (Figure S8). However, no such difference was detected between DEGs and non‐DEGs under DS or 6 h of HS and HD conditions (Figures 4b and S8), suggesting that the association between AS response and transcription activity might vary with different abiotic stresses and time points of stress treatments.

### Comparative analysis of the biological functions regulated at AS and transcription levels

**Figure 5:**
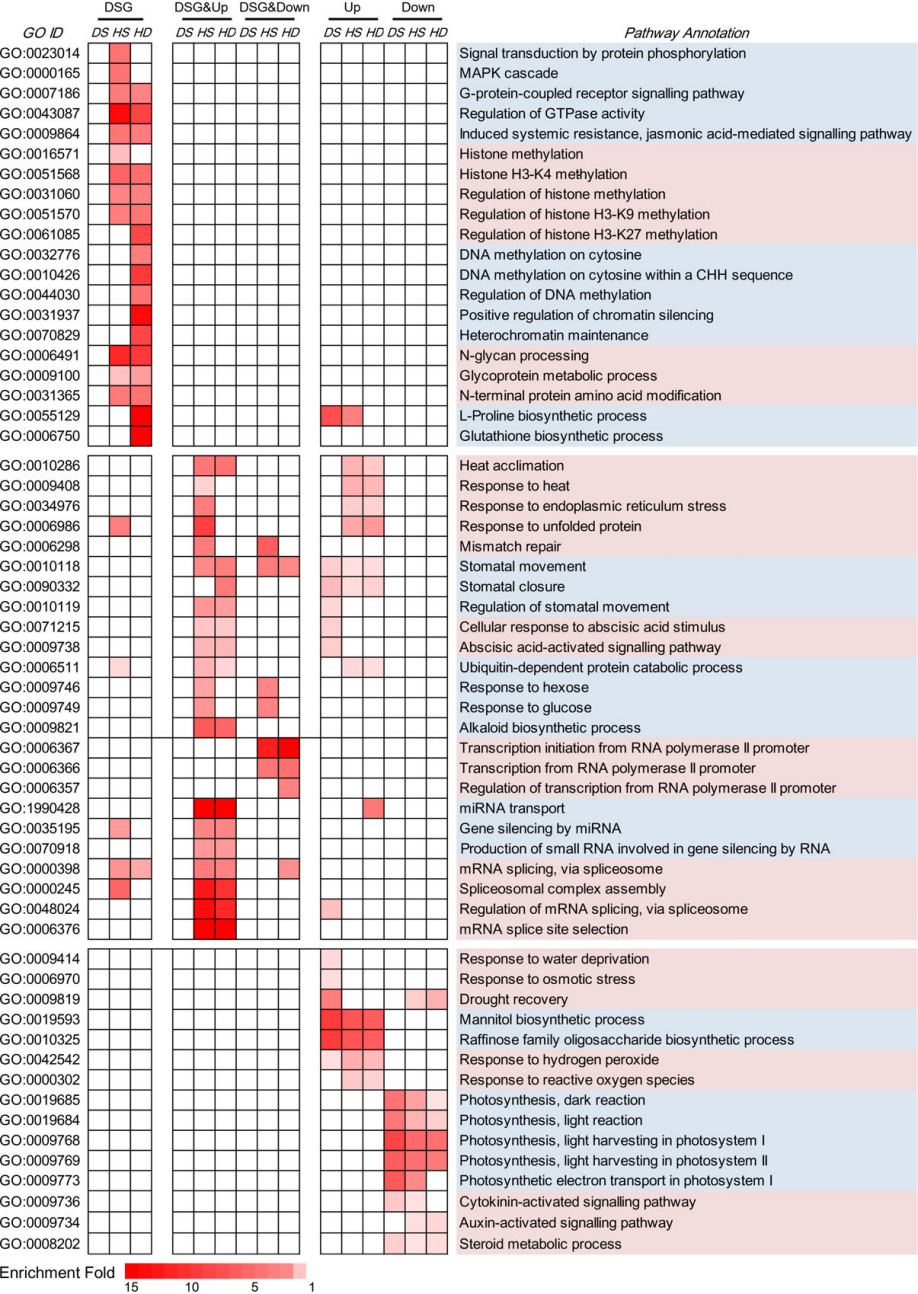
Functional enrichment analysis of DSG‐specific, DEG‐specific and DSG&DEG‐overlapped genes. The enriched Gene Ontology (GO) terms of DSG‐specific, DEG‐specific and DSG&DEG‐overlapped genes are shown in heatmap. DSG: DSG‐specific genes; DSG&Up: genes identified to be both differentially spliced and up‐regulated; DSG&Down: genes identified to be both differentially spliced and down‐regulated; Up: genes only identified to be up‐regulated; Down: genes only identified to be down‐regulated; DS: drought stress; HS: heat stress; HD: heat and drought stress. The colour scale represents enrichment folds of different GO terms, and only significantly enriched terms (*P* < 0.05, enrichment fold ≥1.5) are indicated.

To investigate the biological functions regulated by AS and/or transcriptional modulation, Gene Ontology (GO) enrichment analysis was performed on DSG‐specific, DEG‐specific and DSG&DEG‐overlapped genes, respectively. Consistent with the observation that transcriptional activity rather than AS modulation mainly contributed to the drought response, very few GO terms were found enriching DS‐responsive DSGs, while DS‐responsive DEG‐specific genes were enriched in various drought‐related GO terms, for example ‘response to water deprivation’, ‘response to osmotic stress’, ‘drought recovery’, ‘mannitol biosynthetic process’ and ‘raffinose biosynthetic process’, indicating that drought‐responsive pathways were mainly subjected to transcriptional regulation (Figure 5). By contrast, a set of abiotic stress‐responsive GO terms were significantly overrepresented in DSG&DEG‐overlapped genes under HS and HD conditions (Figure 5), including ‘heat acclimation’, ‘response to heat’, ‘response to ER stress’ and ‘response to unfolded protein’. Besides, ABA signalling‐related GO terms (e.g. ‘cellular response to ABA stimulus’ and ‘ABA‐activated signalling pathway’) and stomatal movement‐related GO terms (e.g. ‘stomatal movements’ and ‘regulation of stomatal movements’) were also significantly overrepresented in DEG&DSG‐overlapped genes after HS and HD treatments (Figure 5). Interestingly, we also found that some important regulatory GO categories exhibited high enrichments in DEG&DSG commonly responsive genes under HS and HD conditions, involving transcriptional regulation (e.g. ‘transcription initiation from RNA Pol II promoter’ and ‘regulation of transcription from RNA Pol II promoter’), RNA‐induced gene silencing (e.g. ‘gene silencing by miRNA’ and ‘production of small RNA involved in gene silencing by RNA’) and pre‐mRNA splicing processes (e.g. ‘spliceosomal complex assembly’, ‘regulation of mRNA splicing’ and ‘mRNA splice site selection’) (Figure 5).

Although various GO terms were found undergoing both AS and transcriptional modulations, the enriched GO terms differed largely between DSG‐ and DEG‐specific genes under HS and HD conditions (Figure 5). Epigenetic process‐related GO terms, such as ‘histone H3K4 methylation’, ‘regulation of histone H3K9/H3K27 methylation’, ‘DNA methylation on cytosine’, ‘regulation of DNA methylation’, ‘chromatin remodelling’ and basic cell signalling processes ‘G‐protein coupled receptor signalling pathway’ and ‘MAPK cascade’ were only overrepresented among DSG‐specific genes in response to HS and HD stresses (Figure 5). In contrast, DEG‐specific genes were enriched in other GO terms, such as ‘response to reactive oxygen species’, ‘photosynthesis’, ‘cytokinin‐activated signalling’ and ‘auxin‐activated signalling’ (Figure 5). Collectively, our results indicated that a wide range of abiotic stress‐responsive pathways were subjected to dual regulation at both transcriptional and AS levels, which also have their preferences in modulating biological functions during HS and HD responses.

## Discussion

### Alternative splicing intensively occurred in response to abiotic stress in wheat seedlings

Plants are subjected to various environmental stresses during their growth and development processes, and to cope with these stresses they have evolved complex mechanisms at both transcriptional and post‐transcriptional levels. However, our current knowledge about transcriptome responses to stress mainly focuses on transcriptional level, while gene regulation at post‐transcriptional levels is much less understood. Alternative splicing (AS) is well demonstrated to play important roles in plant development and stress resistance processes at post‐transcriptional level (Kriechbaumer *et al*., 2012; Nakabayashi *et al*., 2015; Pose *et al*., 2013; Staiger and Brown, 2013), but it is still ambiguous how AS contributes to heat and drought tolerance, especially in wheat. In this study, we performed a genome‐wide survey of AS in wheat seedlings, which revealed a global profiling of AS landscape responsive to DS, HS and HD and suggested that AS may play key roles in conferring tolerance to abiotic stresses in wheat seedlings.

In total, approximately 27% (9312/34 480) of the AS events were identified responsive to DS, HS and/or HD conditions in wheat seedlings, including IR, ES, Alt3′SS and Alt5′SS (Figures 1b and 2a). Several important stress‐responsive genes were found to show conserved alternative splicing patterns in response to stresses among different plant species. For example, *HSFA2* and *DREB2B*, two key abiotic stress regulators, were subjected to AS modulation to confer enhanced stress tolerance in Arabidopsis and rice under HS and DS, and their orthologs were also found exhibiting conserved AS patterns in wheat seedlings (Figure S4) (Cheng *et al*., 2015; Matsukura *et al*., 2010; Sugio *et al*., 2009). In addition, many other well‐documented abiotic stress‐responsive genes, such as *HSP*s, the central players in the thermotolerance response involved in protein folding and degradation, were also subjected to significant AS modulation under DS, HS and HD conditions in wheat seedlings (Figure S3, Data S1) (Jacob *et al*., 2016). Besides, as hexaploid, wheat contains three homeologous subgenomes, we found homeologous triplets exhibiting partitioned AS responses under DS, HS and HD conditions, which indicated subfunctionalization or neofunctionalization of homeologous genes in terms of alternative splicing during wheat polyploidization history (Figure S2). Therefore, AS can diversify transcriptome reprogramming in response to abiotic stresses in wheat, which may serve as an important molecular mechanism for plant to adapt to hostile environments, although further experimental investigations are needed to draw explicit conclusions on their biological relevance. However, considering limited number of wheat tissues and treatments, AS genes might be partly overlooked in this study because of tissue‐ or stress‐specific gene expression patterns. Additionally, the identification of AS genes based on RNA‐Seq analysis could miss events that lack junction supporting reads due to sequencing depth. Taken together, it is reasonable to hypothesize that the number of AS genes in wheat is underestimated due to technical issues and the inherently dynamic nature of AS in plants.

Up to date, the potential molecular mechanisms underlying AS responses to abiotic stresses are still ambiguous in wheat. It is well documented that UA richness of introns and CG richness of exon contribute to the splicing site recognition of *Ser/Arg‐rich (SR)* genes, the key regulators of alternative splicing, whose abundance and activity determines the AS profiles of their target genes (Reddy and Ali, 2011), and AS pattern of *SR* genes is affected by abiotic stresses including temperature (Gulledge *et al*., 2012; Lazar and Goodman, 2000). In this study, there were more *SR* genes responsive to HS and HD stress treatments than to DS at both transcriptional and splicing levels (Figure S5). The contrasting number of stress‐responsive *SR* genes and their differential responses to HS, HD and DS are consistent with the global AS landscapes under these three stress conditions, which might explain why enhanced AS variations were observed under HS and HD compared with DS (Figure 2a). In addition, DNA methylation is one of several epigenetic mechanisms that is known to regulate gene transcription and was recently found to be involved in splicing modulation *via* differential methylation frequency in exons and introns (Naftelberg *et al*., 2015; Wang *et al*., 2016). Previous study documented that DNA methylation landscape varied in response to different temperatures in wheat (Gardiner *et al*., 2015). We found that more DNA methylation‐related genes were found to be differentially expressed under HS (33) and HD (42) compared with DS (14) (Data S4). And there were eight and nine DNA methylation‐related genes exhibiting alternative splicing responsive to HS and HD, whereas no alternatively spliced DNA methylation‐related gene was observed under DS (Figure 5, Data S1). All these data suggested that DNA methylation might also be involved in AS regulation in wheat.

### The combination of heat and drought stress can induce specific AS responses

In natural environment, plants are often subjected to multiple stresses, for example heat and drought combined stress, which can cause great disadvantageous effects on plant growth and yield production (Pradhan *et al*., 2012; Prasad *et al*., 2011; Shah and Paulsen, 2003). Although studies on abiotic stress response have greatly advanced in recent years, how plants orchestrate responses at different levels to cope with heat and drought combined stress is still ambiguous. Several lines of evidences indicated that, rather than being simply additive, the way how plants respond to combined stresses occurred in the field is largely distinct from individual ones (Johnson *et al*., 2014; Pradhan *et al*., 2012; Rizhsky *et al*., 2004; Szucs *et al*., 2010). Previous transcriptome analysis of wheat seedling leaves subjected to DS, HS and HD demonstrated that about 17%–36% DEGs showed specific responses to HD in wheat, suggesting that stress combination requires unique acclimation responses that cannot be induced by individual stress alone (Liu *et al*., 2015). Consistently, in the present study, we found that 963 AS events significantly responded to the combination of heat and drought stress but barely changed under individual HS and DS, suggesting that these HD‐specific AS responses might be caused by the interaction between drought and heat responses (Clusters 10–12 in Figure 2c). Interestingly, several previously reported stress‐responsive genes also exhibited HD‐specific AS response, for example *TNO1* (involved in salt tolerance), *WDR5A* (involved in drought tolerance), *AL5* (involved in salt, drought and freezing tolerance) and *DBP1* (involved in virus resistance) (Figure S3, Data S1) (Castello *et al*., 2011; Kim and Bassham, 2011; Liu *et al*., 2017; Wei *et al*., 2015). Moreover, some GO terms, such as ‘glutathione biosynthetic process’ and ‘DNA methylation’, significantly enriched HD‐specific AS genes, indicating that these processes tended to be responsive to the combination of heat and drought stress at AS level (Figure 5). Conclusively, plant requires specific responses at both transcriptional and AS levels to adapt to heat and drought combined stress which usually occurred in the field.

### AS and transcriptional modulations cooperate to fine‐tune HS and HD responses in wheat

Previous studies have investigated the AS responses under heat and drought stress in different plant species; however, a parallel comparison of AS responses induced by these two conditions has not yet been reported (Chang *et al*., 2014; Jiang *et al*., 2017; Keller *et al*., 2016; Thatcher *et al*., 2016). Our results indicated that much stronger AS responses were induced by HS and HD than by DS, while the transcriptional responses were found comparable under these three conditions, suggesting that DS response was mainly due to transcriptional modulation, whereas HS and HD responses could be attributed to both AS (DSGs) and transcriptional regulation (DEGs) in wheat seedling leaves (Figures 2a, 4a and S6).

Moreover, it is proposed that AS and transcriptional regulation are two parallel processes functioning independently in response to environmental stresses in plant, and very little overlap of DSGs and DEGs was found in response to salt stress, heat stress, Fe deficiency as well as biotic stress in Arabidopsis, *Physcomitrella patens* and *Nicotiana attenuata* (Chang *et al*., 2014; Ding *et al*., 2014; Li *et al*., 2013; Ling *et al*., 2015). These were concordant with our observation in wheat seedlings under DS that only 12% of DSGs were also subjected to transcriptional modulation (Figure 4a). However, our observation under HS and HD was contradictory to the previous reports, which might result from different stresses, various species/organs or even developmental stages (Figure 4a). Here, we found that up to 40%–42% DSGs were overlapped with DEGs under HS and HD conditions (Figure 4a). This was concordant with further GO enrichment analyses, which demonstrated that various abiotic stress‐responsive GO terms were significantly overrepresented among DSG&DEG‐overlapped genes under HS and HD conditions, including ‘heat acclimation’, ‘response to ER stress’, ‘regulation of stomatal movements’ and ‘ABA‐activated signalling pathway’, whereas very few GO terms were enriched with DSG&DEG‐overlapped genes under DS condition (Figure 5). Collectively, our results strongly suggested that the coordination of AS and transcriptional regulation was required to orchestrate the stress responses to better cope with HS and HD in wheat.

Previous studies in animals and yeasts have demonstrated that pre‐mRNA splicing was cotranscriptionally regulated and the AS of genes can be affected by their transcriptional activity and chromatin modifications (Jimeno‐Gonzalez *et al*., 2015; Naftelberg *et al*., 2015; Saldi *et al*., 2016). We observed that DEGs showed stronger AS responses than non‐DEGs after 1 h of HS and HD treatments, suggesting that the cotranscriptional regulation of AS might also exist in plants (Figures 4b, S7 and S8). However, a substantial number of DSGs did not exhibit significant transcriptional changes and vice versa (Figure 4), suggesting that non‐DEGs might also play important roles in stress tolerance and the interaction between transcriptional and AS modulations is complex in plant, which need further investigation.

### Modulation of AS can be a possible strategy for crop stress tolerance improvement

Alternative splicing serves to diversify plant's transcriptome and proteome by producing more than one mature transcript from a single pre‐mRNA, which contributes to the plasticity and adaptation of plant to stress conditions (Staiger and Brown, 2013). Being sessile nature, crops suffer a wide range of stresses which lead to significant yield loss. To improve crop adaptation to hostile environments *via* biotechnological approaches, the prerequisite is to identify genes responsible for the determination of stress tolerance. Previous efforts have been made to identify candidate genes for crop improvement in response to hostile environments, which indeed dramatically enhances stress tolerance in many plant species (Chen *et al*., 2008; Ogawa *et al*., 2007). However, accompanying side effects often occurred extensively to transgenic plants, because constitutional expression of a stress‐responsive candidate gene usually results in unfavourable phenotypes such as dwarfism and aberrant morphology (Matsukura *et al*., 2010; Ogawa *et al*., 2007).

Yet, alternative splicing might provide a novel strategy for regulating the environmental fitness of plants. A proportion of candidate AS genes may play an important role in plant development with one splicing isoform, whereas its alternative isoform was only induced under stressed conditions, which then participated in modulating responses to environmental stresses, such as *HSFA2* and *DREB2* (Figure S4) (Cheng *et al*., 2015; Matsukura *et al*., 2010; Sugio *et al*., 2009). Therefore, proper manipulation of stress‐responsive AS genes could achieve ‘one stone two birds’ that one transcript assists crop development under normal condition, whereas the other one helps to enhance stress tolerance under hostile conditions. Moreover, it is reported that plant *SR* genes possess development‐stage or stress‐specific regulation of alternative splicing, providing us opportunities to improve crop's stress tolerance by manipulating stress‐responsive *SR* genes (Reddy and Ali, 2011). Under this circumstance, the AS profiles of a set of downstream genes can be well balanced between normal and stress conditions, which might reduce side effects on plant development. Conclusively, AS is one of major molecular mechanisms that plants fine‐tune their gene interaction networks, and can serve as a potential strategy to regulate gene functioning and improve crop tolerance to abiotic stresses.

## Experimental procedures

### Plant materials, stress treatments and RNA sequencing

RNA‐Seq data used in this study were generated in our previous reports (NCBI SRA database: SRP045409) using the following procedure (Liu *et al*., 2015). TAM107 is a leading wheat variety during late 1980s and early 1990s in western Kansas, which was released by Texas A&M University in 1984. And it developed a reputation for both heat and drought tolerant (Wheat Genetics Resource Center, Kansas). Seeds of ‘TAM 107’ were surface‐sterilized in 1% sodium hypochlorite for 20 min, rinsed in distilled water, cultivated in water and grown in a growth chamber with 22 °C/18 °C (day/night), 16 h/8 h (light/dark) and 50% humidity for a week. Then, the seedlings were subjected to heat stress (40 °C), drought stress (20% (m/V) PEG‐6000) and combined heat and drought stress (40 °C and 20% PEG‐6000) for 1 h and 6 h, respectively. Drought stress was applied by replacing water with 20% PEG solution and roots were totally covered by PEG solution (Dhanda *et al*., 2004). Heat stress was applied by moving the plants to another growth chamber with 40 °C temperature. All experiments were performed in parallel and seedlings in normal growth condition (22 °C, well watered) were taken as control. Five independent biological replicates were employed, with two for sequencing and the other three for experimental verification. Leaves were collected separately at 1 h and 6 h after stress treatment and frozen immediately in the liquid nitrogen.

Total RNA from leaf tissues was extracted using TRIzol reagent (Invitrogen Carlsbad, California, US), according to the manufacturer's instructions. RNA concentration was measured using NanoDrop 2000 spectrophotometer (ND‐2000, ThermoFisher Scientific, Inc. Waltham, MA, US). RNA integrity was assessed on an Agilent 2100 Bioanalyzer (Agilent Technologies, Inc., Santa Clara, California, US). Paired‐end (PE) sequencing libraries with average insert size of 200 bp were prepared with TruSeq RNA Sample Preparation Kit v2 (Illumina, San Diego, CA) and sequenced on HiSeq2000 (Illumina) according to the manufacturer's standard protocols. Raw data obtained from Illumina sequencing were processed and filtered using Illumina pipeline (http://www.Illumina.com) to generate FastQ files. Finally, approximately 184.3G high‐quality 100‐bp pair‐end reads were generated from 14 libraries (Table S1).

### Reads mapping and transcript structure assembly

An overview of bioinformatics pipeline used for data analysis is shown in Figure S1. High‐quality RNA‐Seq reads from each library were aligned to wheat reference genome (IWGSC1.0 wheat reference genes from EnsemblPlants) using Tophat2 (version 2.0.14) with stringent parameters ‘–b2‐L 15 –segment‐mismatches 1 –read‐gap‐length 1 –b2‐score‐min L,‐0.6,‐0.3 –no‐coverage‐search ‐N 1 –read‐edit‐dist 2 –read‐realign‐edit‐dist 2′, which only allow one mismatch for each alignment (Kim *et al*., 2013). Next, reads aligned to more than one chromosome position were excluded by custom Perl scripts to minimize artefacts from ambiguous mapping.

To obtain a comprehensive alternative splicing landscape of wheat seedlings, filtered alignments of each sample were used to assemble transcript structures by Cufflinks (version. 2.2.1) with parameters ‘–pre‐mrna‐fraction 0.1 –3‐overhang‐tolerance 400 –min‐frags‐per‐transfrag 15 ‐u’ (Trapnell *et al*., 2012). And, the IWGSC gene reference annotation was supplied as additional assembly information (parameter ‘‐g’). Novel transcript structures supported by fewer than 15 aligned RNA‐Seq fragments were filtered out to ensure the assemble accuracy. Then, individual predicted transcript models were combined with Cuffmerge (version. 2.2.1), which takes the cufflinks output structures and creates a nonredundant set of transcript structures by merging it with IWGSC gene reference annotation (Trapnell *et al*., 2012).

### AS detection and stress‐responsive AS events identification

As Figure 1a shows, every AS event refers to two types of splice isoforms which are represented by different splice junctions (for ES, Alt3′SS and Alt5′SS) or exon–intron junctions (for IR). To identify AS events in wheat seedlings, exon–exon and exon–intron junction information was extracted from GTF file generated by Cuffmerge. Then, custom Perl scripts were used to count the number of supporting reads for each detected junction based on BAM and BED files generated by Tophat2. To remove the AS events potentially predicted by mapping error, only junctions supported by at least five uniquely mapped reads in at least one sample were considered in further analysis. All AS events in wheat seedlings, consisting of IR, ES, Alt3′SS and Alt5′SS, were identified based on these filtered junctions by custom Perl scripts using the methods described by Brooks *et al*. (2011).

To identify stress‐responsive AS events, Fisher's exact tests in R (http://www.r-project.org/) were performed on the comparison of junction reads counts supporting different splice isoforms between control and stress conditions. Next, isoform expression percentage (IEP) of each AS event was calculated as follows: junction reads counts_isoform1_/junction reads counts_(isoform1+isoform2)_. And the IEP change between control and each stress condition was calculated as |IEP_control_−IEP_stress_|*100%, which reflected AS pattern changes in response to stress treatments (Brooks *et al*., 2011). Finally, AS events with IEP change more than 30% and FDR‐corrected *P* values (Benjamini and Hochberg's method) <0.05, and have the same pattern changes in two biological replicates after each stress treatment was identified as stress‐responsive AS events (Chang *et al*., 2014; Ling *et al*., 2015).

### Differential gene expression analysis

FPKM (Fragments Per Kilobase of transcript per Million mapped fragments) computation and differential gene expression analysis were performed by using Cuffquant (version. 2.2.1) and Cuffdiff (version. 2.2.1) programs in TUXEDO workflow with parameters ‘‐u –min‐reps‐for‐js‐test 2 ‐M –min‐alignment‐count 15 –compatible‐hits‐norm’ (Trapnell *et al*., 2012). Genes with more than twofold changes and a false discovery rate (FDR)‐adjusted *P*‐value <0.05 were considered as differentially expressed genes (DEGs, Figures 4a and S6).

### Functional annotation and gene ontology enrichment analysis

All wheat reference genes were aligned against Arabidopsis protein database using local Blast search. Arabidopsis gene function information was obtained from TAIR (http://www.arabidopsis.org/). And the best hits with e‐value less than 1e−10 and identical animal acids more than 40 were retained for functional annotation. Gene Ontology (GO) annotations were obtained from Gene Ontology Consortium (http://www.geneontology.org/), which were used to annotate wheat genes. The significant overrepresented GO terms were determined by Fisher's exact test *P*‐value <0.05 and enrichment fold ≥1.5 compared with whole‐genome background.

### RT‐PCR validation of AS events

Total RNA treated with DNase I were reverse‐transcribed with oligo‐dT primers using Reverse Transcriptase M‐MLV (TaKaRa, Kusatsu, Shiga, Japan) following the manufacturer's instructions. RT‐PCR was performed in a 10 μL reaction volume and primer pair for each gene was designed to amplify both two splice variants (isoforms 1 and 2) in a single reaction. The primers used in RT‐PCR analysis are listed in Table S2.

## Conflict of interest

The authors declare that they have no conflict of interests.

## Supporting information


**Figure S1** Pipeline of alternative splicing analysis.
**Figure S2** Comparison of differentially spliced homeologous triplets.
**Figure S3** AS profiles of previously reported stress responsive genes under DS, HS and HD conditions.
**Figure S4** Expression analysis of the AS isoforms of *TaHSFA2* and *WDREB2* in response to DS, HS and HD conditions.
**Figure S5** Expression and AS analysis of *SR* genes in response to DS, HS and HD treatments.
**Figure S6** Number of differentially expressed genes (DEGs) identified under each stress conditions.
**Figure S7** Comparison of the proportion of DSGs in DEGs and non‐DEGs for A, B and D subgenomes under each stress conditions.
**Figure S8** Distribution of IEP changes among up‐regulated, down‐regulated and non‐DEGs under each stress conditions.

**Table S1** Statistical summary of reads mapping.
**Table S2** Primers used in experimental validation of stress responsive AS.

**Data S1** Summary of stress responsive AS events under DS, HS and HD conditions.

**Data S2** PTC prediction of stress responsive AS events.

**Data S3** Clustering of stress responsive AS events based on their AS pattern changes after DS, HS and HD treatments.

**Data S4** Lists of differentially expressed genes identified under DS, HS and HD conditions.
