# Supplementary material for: Global profiling of alternative splicing landscape responsive to drought, heat and their combination in wheat (Triticum aestivum L.)
Source: Plant Biotechnol J. 2017 Sep 20;16(3):714–26. doi: 10.1111/pbi.12822 (PMC5814593; doi:10.1111/pbi.12822)
Supplement: Supplementary file 1 — Figure S1 Pipeline of alternative splicing analysis. Figure S2 Comparison of differentially spliced homeologous triplets. Figure S3 AS profiles of previously reported stress responsive genes under DS, HS and HD conditions. Figure S4 Expression analysis of the AS isoforms of TaHSFA2 and WDREB2 in response to DS, HS and HD conditions. Figure S5 Expression and AS analysis of SR genes in response to DS, HS and HD treatments. Figure S6 Number of differentially expressed genes (DEGs) identified under each stress conditions. Figure S7 Comparison of the proportion of DSGs in DEGs and non‐DEGs for A, B and D subgenomes under each stress conditions. Figure S8 Distribution of IEP changes among up‐regulated, down‐regulated and non‐DEGs under each stress conditions. [file PBI-16-714-s005.doc]

**Supporting Information: Figure S1-S8**


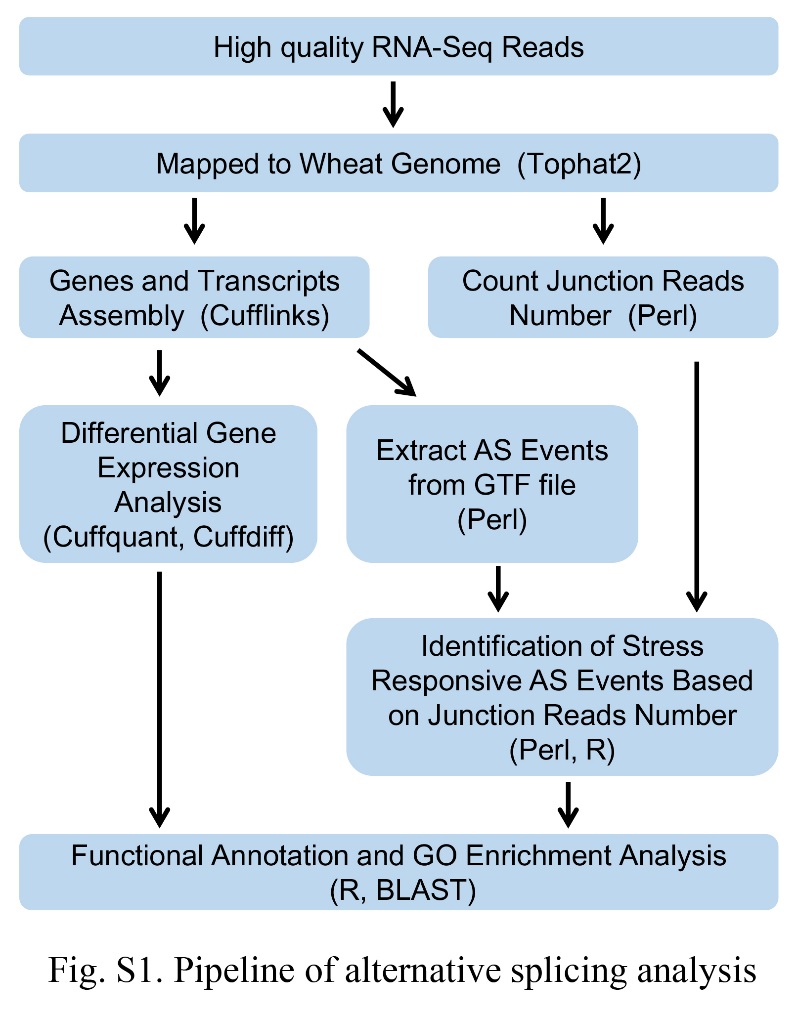


**Figure S1.** Pipeline of alternative splicing analysis.


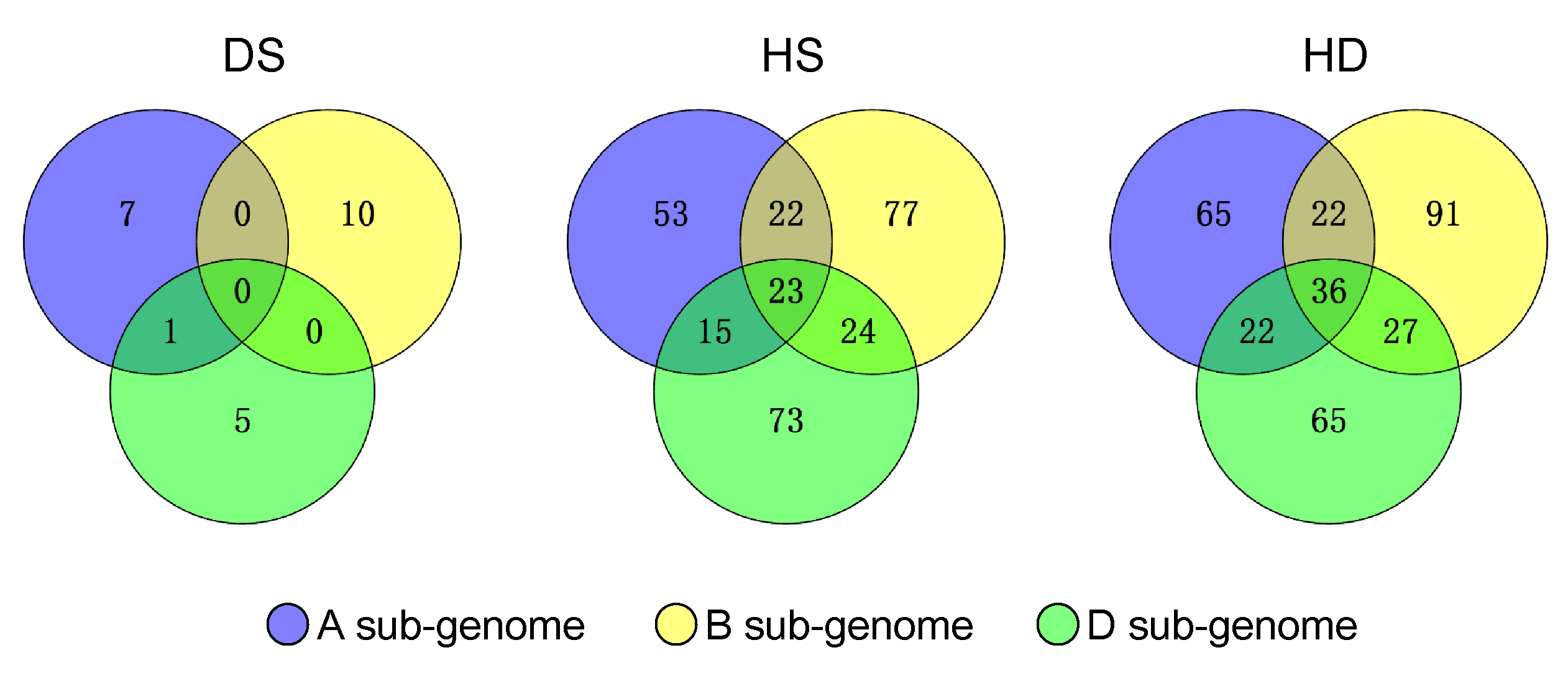


**Figure S2**. Comparison of differentially spliced homeologous triplets.

Homeologous triplet was defined as gene loci that had exactly one representative member from each subgenomes. Triplets with all three homeologous genes being expressed were retained for this analysis (3,499 triplets, 3,499×3=10,497 homeologs). Among these triplets, a total of 23, 287 and 328 triplets were identified to be differentially spliced (with at least one homeolog being differentially spliced) under DS, HS and HD conditions, respectively. And A, B and D homeologs of these differentially spliced triplets were compared by venn diagrams.


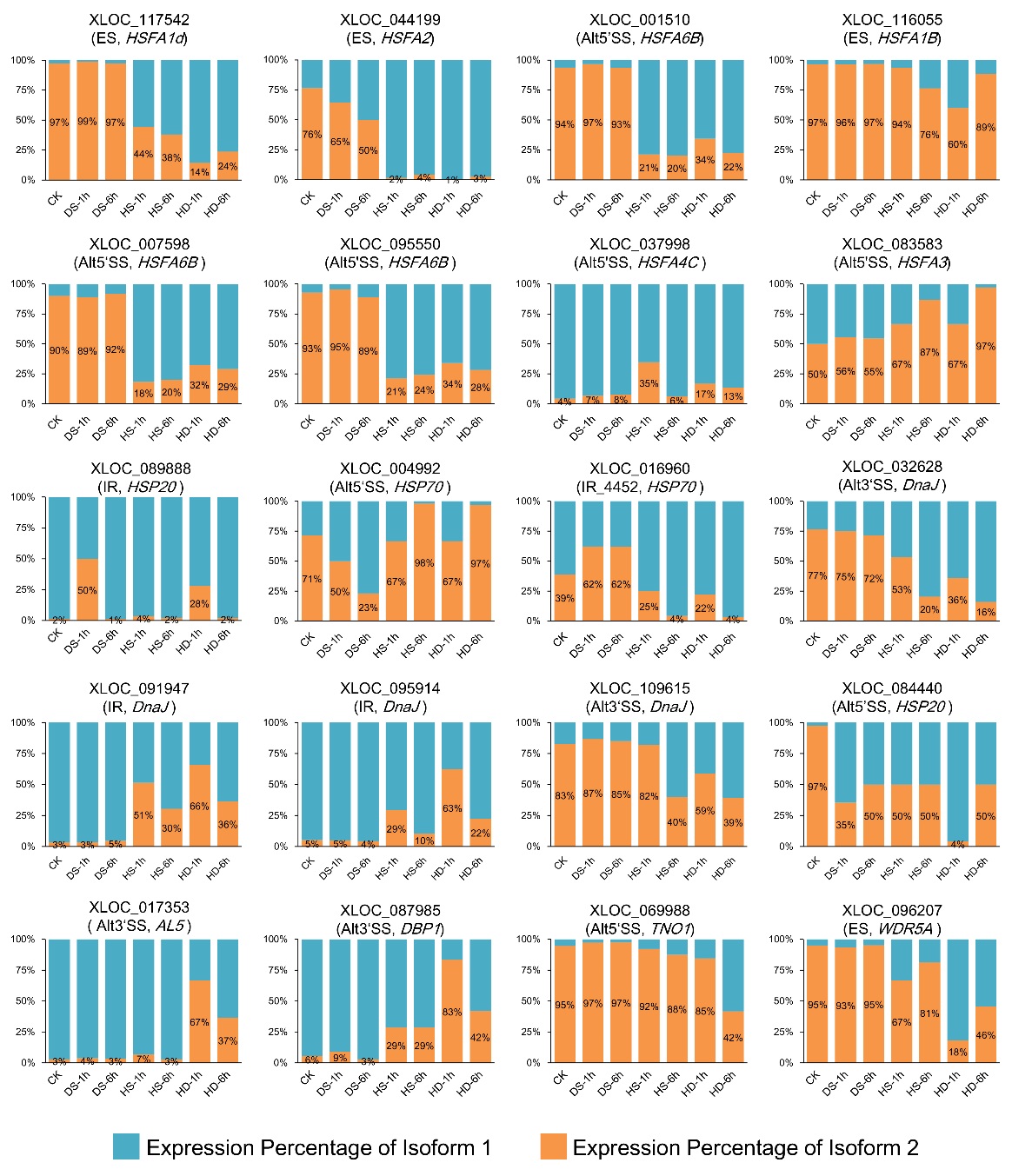


**Figure S3.** AS profiles of previously reported stress responsive genes under DS, HS and HD conditions.

The bar charts showed relative expression level of alternatively spliced isoform 1 (blue) and isoform 2 (yellow) of AS genes under DS, HS and HD revealed by RNA-Seq. The numbers in each yellow bars indicate the expression percentage of isoform 2.


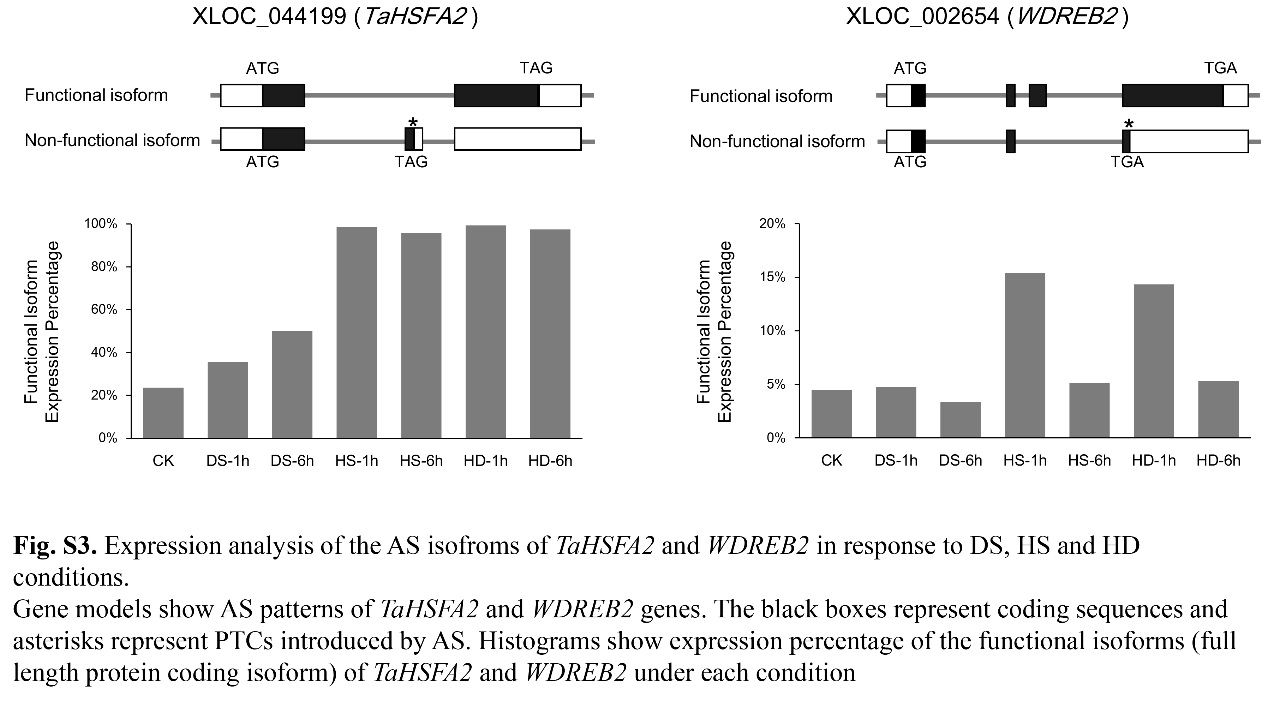


**Figure S4.** Expression analysis of the AS isoforms of *TaHSFA2* and *WDREB2* in response to DS, HS and HD conditions.

Gene models show AS patterns of *TaHSFA2* and *WDREB2* genes. The black boxes represent coding sequences and asterisks represent PTCs introduced by AS. Histograms show expression percentage of the functional isoforms (full length protein coding isoform) of *TaHSFA2* and *WDREB2* under each condition.


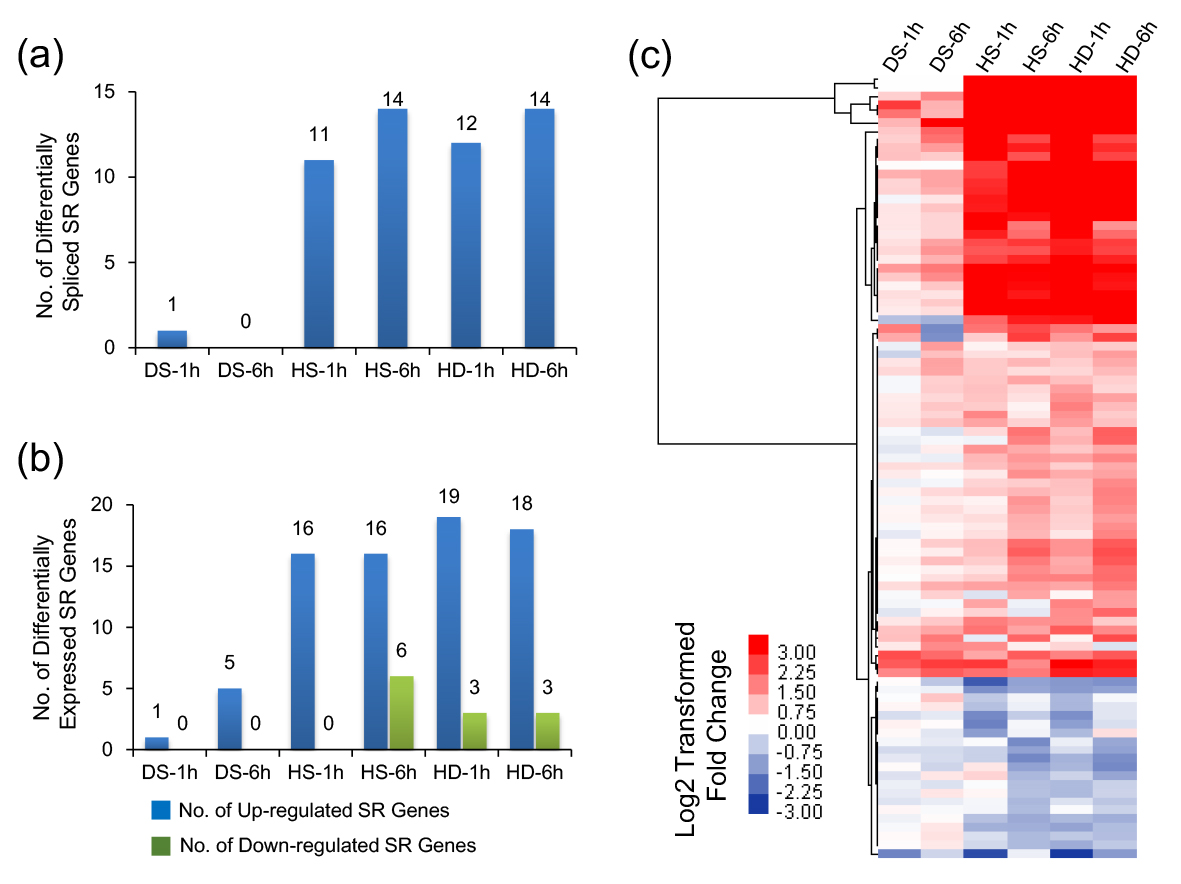


**Figure S5.** Expression and AS analysis of *SR* genes in response to DS, HS and HD treatments.

1. The number of differentially spliced *SR* genes identified after 1h and 6h of DS, HS and HD treatments.
2. The number of differentially expressed *SR* genes identified after 1h and 6h of DS, HS and HD treatments.
3. Clustering analysis of differentially expressed *SR* genes under 1h and 6h of DS, HS and HD conditions. The fold-change of *SR* gene expression level after each stress treatment was calculated and Log2 transformed. Up-regulated (red) and Down-regulated (blue) SR genes were represented by different color scale in heatmap.


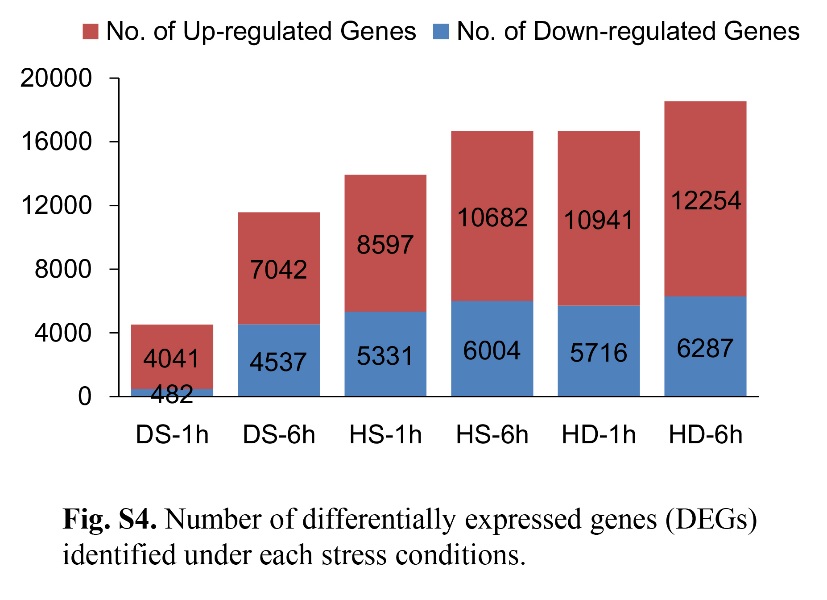


**Figure S6.** The number of differentially expressed genes (DEGs) identified under DS, HS and HD conditions.


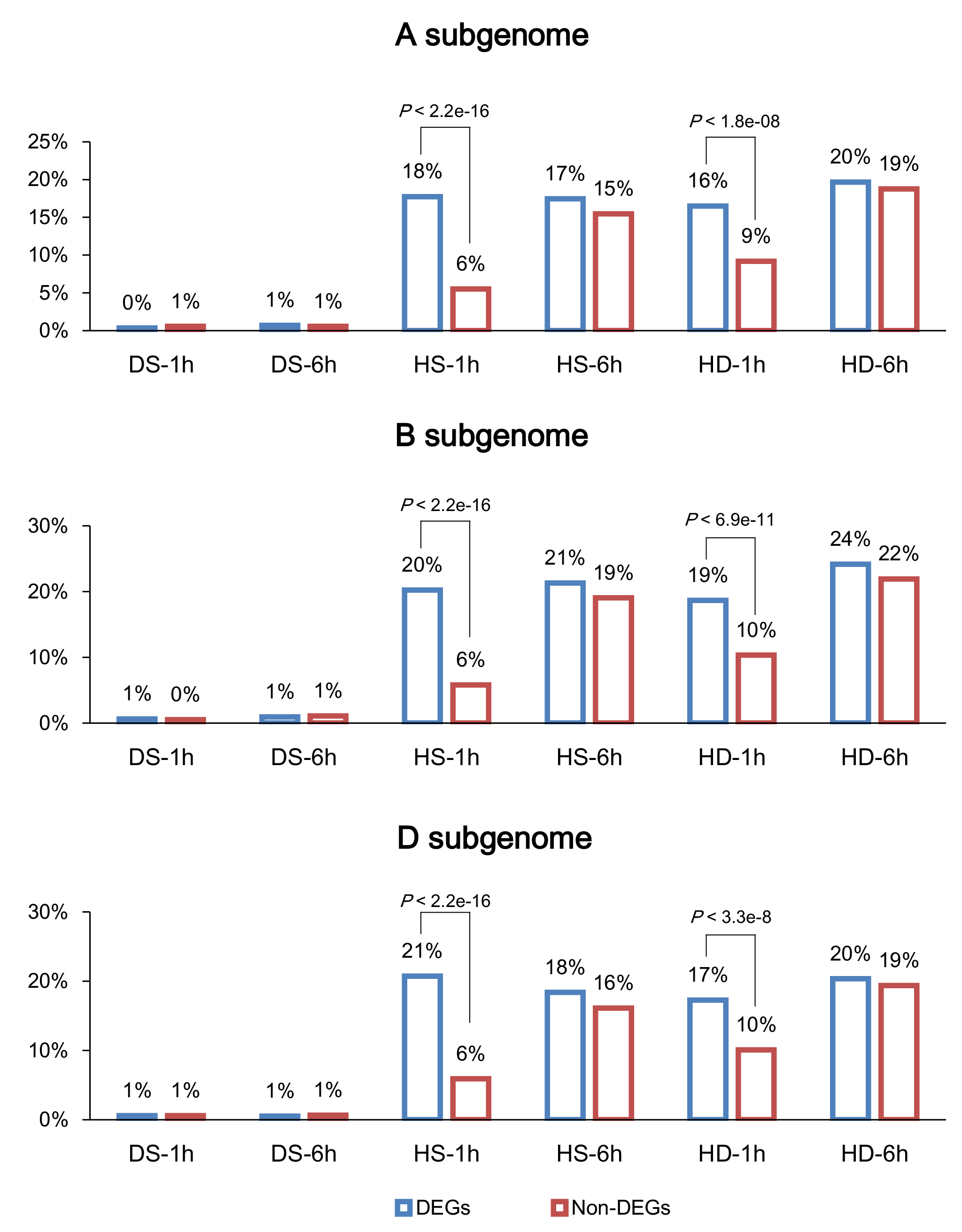


**Figure S7.** Comparison of the proportion of DSGs in DEGs and non-DEGs for A, B and D subgenomes under each stress condition.

Only genes with more than 10 junction supporting reads were considered for the analysis. The percentage of DSGs in DEGs is much higher than in non-DEGs under HS-1h and HD-1h, and the *p*-value indicated significance level based on Fisher’s Exact Test.


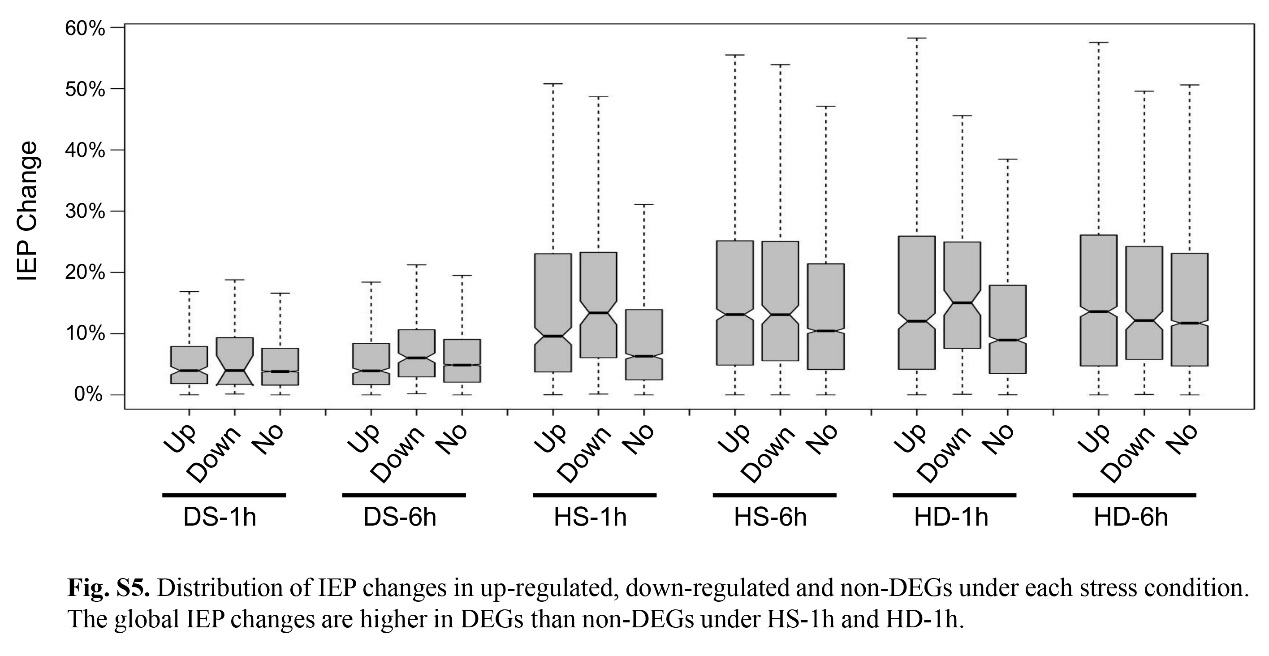


**Figure S8.** Distribution of IEP changes among up-regulated, down-regulated and non-DEGs under each stress condition.

The global IEP change is higher in DEGs than non-DEGs under HS-1h and HD-1h.
