# Supplementary material for: Global profiling of alternative splicing landscape responsive to drought, heat and their combination in wheat (Triticum aestivum L.)
Source: Plant Biotechnol J. 2017 Sep 20;16(3):714–26. doi: 10.1111/pbi.12822 (PMC5814593; doi:10.1111/pbi.12822)
Supplement: Supplementary file 2 — Table S1 Statistical summary of reads mapping. Table S2 Primers used in experimental validation of stress responsive AS. [file PBI-16-714-s004.docx]

**Supporting Information: Table S1-S2**

**Table S1.** Statistical summary of reads mapping.

| Samples | No. of High Quality Reads | No. of Uniquely Mapped Reads | %. of Uniquely Mapped Reads |
| --- | --- | --- | --- |
| CK-1 | 81,331,219 | 45,632,172 | 56% |
| CK-2 | 76,134,375 | 41,326,867 | 54% |
| DS1h-1 | 68,627,235 | 38,752,817 | 56% |
| DS1h-2 | 75,990,953 | 45,694,095 | 60% |
| DS6h-1 | 63,466,609 | 36,874,847 | 58% |
| DS6h-2 | 73,803,033 | 43,795,126 | 59% |
| HS1h-1 | 66,201,326 | 38,700,700 | 58% |
| HS1h-2 | 51,678,457 | 33,112,888 | 64% |
| HS6h-1 | 76,714,383 | 50,576,106 | 66% |
| HS6h-2 | 67,490,014 | 44,028,819 | 65% |
| HD1h-1 | 53,916,631 | 31,323,842 | 58% |
| HD1h-2 | 55,730,935 | 31,914,997 | 57% |
| HD6h-1 | 54,038,225 | 32,906,359 | 61% |
| HD6h-2 | 56,455,411 | 34,338,844 | 61% |

**Table S2.** Primers used in experimental validation of stress responsive AS.

| **Primers** | **Sequences (5'-3')** |
| --- | --- |
| XLOC_106140-F | CTCCCTACAGTGCTAGTCCTTG |
| XLOC_106140-R | GGAAAGCTACGAAGAGTAGATGAC |
| XLOC_049104-F | CTGTTCCTTTTAGCGCCTCA |
| XLOC_049104-R | GCGTGACAGTTGCCAAGATTC |
| XLOC_002075-F | TGTCATCAATAGAGAGGCAAAGATC |
| XLOC_002075-R | GTCAACGAGTAAGATTTCAGAGGAG |
| XLOC_091947-F | TAGCGGTAGTCGTCAAACTG |
| XLOC_091947-R | CCACTGCCACTATTATCACCAT |
| XLOC_044199-F | CAGCAACTTCTCCAGCTTCGTCC |
| XLOC_044199-R | CCTCTGCGAGTAAGGCGTTCTTG |
| XLOC_117542-F | CTACTGCAAGGACGAGGAGTAG |
| XLOC_117542-R | AGGGCAGGACTGTCAGATGG |
| XLOC_116055-F | ACAACAGCATATACACCGAGCGAGTG |
| XLOC_116055-R | AGAGCAGGACTGTCAGATGGAAGCA |
| XLOC_001510-F | CTCAGTGAACCTGGAATGAACG |
| XLOC_001510-R | TCCATCAAGCGACGCAGCA |
